# Parental Age and Stress in Caregivers of Individuals with Angelman Syndrome: Insights from the LADDER Cohort

**DOI:** 10.1093/geroni/igaf122.3989

**Published:** 2025-12-31

**Authors:** Sean Halpin, Sarah Potter, Wen-Hann Tan, Anjali Sadhwani, Batsheva Friedman, Anne Wheeler

**Affiliations:** RTI International, Decatur, Georgia, United States; RTI International, Research Triangle, North Carolina, United States; Harvard Medical School, Boston, Massachusetts, United States; Harvard Medical School, Boston, Massachusetts, United States; Harvard Medical School, Boston, Massachusetts, United States; RTI International, Research Triangle Park, North Carolina, United States

## Abstract

Angelman syndrome (AS) is a rare neurogenetic disorder characterized by severe developmental delay including minimal speech and motor impairments. As individuals with AS grow older, many parents provide intensive support into their own later life. Using data from the Angelman Syndrome Natural History Study, via the Linking Angelman and Dup15q Data for Expanded Research (LADDER) Database, we examined how maternal age relates to stress domains. Our sample included 268 participants (591 observations), with maternal ages of 23–64 years (baseline mean = 36, SD = 7.1). Mixed-effects REML regression models assessed associations between child and maternal age and 16 stress domains from the Parenting Stress Index. Maternal age was significantly linked to increased stress in child adaptability (β = 0.574, p = .014), reinforcement (β = 0.566, p = .008), demandingness (β = 0.692, p < .001), mood (β = 0.864, p < .001), isolation (β = 0.653, p = .005), and total stress (β = 0.548, p = .012). These effects held after adjusting for child age, which also correlated with stress in adaptability (β = 1.007, p < .001) and mood (β = 1.269, p < .001). Older caregivers face compounding stress from aging children’s behavioral challenges and their own age-related vulnerabilities. Age-responsive interventions are needed to support long-term caregivers and guide future research into late-life caregiving in rare neurodevelopmental disorders.

